# RNA-Centric Approaches to Profile the RNA–Protein Interaction Landscape on Selected RNAs

**DOI:** 10.3390/ncrna7010011

**Published:** 2021-02-15

**Authors:** André P. Gerber

**Affiliations:** Department of Microbial Sciences, School of Biosciences and Medicine, Faculty of Health and Medical Sciences, University of Surrey, Guildford GU2 7XH, UK; a.gerber@surrey.ac.uk

**Keywords:** RNA–protein interaction, non-coding RNA, RNA aptamer, antisense oligonucleotides, affinity isolation, CRISPR/Cas9, mass-spectrometry

## Abstract

RNA–protein interactions frame post-transcriptional regulatory networks and modulate transcription and epigenetics. While the technological advances in RNA sequencing have significantly expanded the repertoire of RNAs, recently developed biochemical approaches combined with sensitive mass-spectrometry have revealed hundreds of previously unrecognized and potentially novel RNA-binding proteins. Nevertheless, a major challenge remains to understand how the thousands of RNA molecules and their interacting proteins assemble and control the fate of each individual RNA in a cell. Here, I review recent methodological advances to approach this problem through systematic identification of proteins that interact with particular RNAs in living cells. Thereby, a specific focus is given to in vivo approaches that involve crosslinking of RNA–protein interactions through ultraviolet irradiation or treatment of cells with chemicals, followed by capture of the RNA under study with antisense-oligonucleotides and identification of bound proteins with mass-spectrometry. Several recent studies defining interactomes of long non-coding RNAs, viral RNAs, as well as mRNAs are highlighted, and short reference is given to recent in-cell protein labeling techniques. These recent experimental improvements could open the door for broader applications and to study the remodeling of RNA–protein complexes upon different environmental cues and in disease.

## 1. Introduction

RNA–protein interactions are the nodes in a complex regulatory network that controls the fate of each RNA expressed in a cell. On the one hand, recent advances in RNA sequencing (RNA-seq) and computational techniques have dramatically expanded the repertoire of cellular RNAs, realizing that 80–90% of the eukaryotic genome is transcribed into RNA. Thereby, only 1–2% of the genome serves as a template for protein synthesis, while the majority of the genome is pervasively transcribed into non-coding RNAs, a large fraction of them as long non-coding RNAs (lncRNAs), which are defined as transcripts of more than 200 nucleotides without evident protein coding functionality [1,2]. On the other hand, bioinformatics and recent methodological advances combined with sensitive mass-spectrometry (MS) suggests that 5–10% of the cellular proteome can interact with RNA [3,4,5]. These RNA-binding proteins (RBPs) either bind to distinct sites or interact promiscuously with their RNA targets, thereby forming ribonucleoprotein (RNP) complexes that combinatorically control the fate of RNA [6]. Canonical RBPs bear one or several characteristic RNA-binding domains, such as the RNA-recognition motif (RRM), the hnRNPK homology (KH) domain, double-stranded RNA-binding motif (DSRM), or zinc-finger (ZnF) domains that determine the specificity and affinity towards the RNAs [7,8]. Nevertheless, unconventional RBPs that lack such domains but have other well-characterized cellular functions have also been discovered, including metabolic enzymes, heat shock proteins, kinases, as well as transcription regulators [5,9,10]. Overall, the wealth of RNAs and their protein partners suggests the formation of a highly structured network that assumingly fulfils additional functions beyond the control of gene expression. The RNA–protein network likely integrates signals from diverse sources to eventually coordinate the structural and functional units required for cell viability. Thus, understanding how and where RNA–protein interactions take place in cells is key to understand the link between RNA biology and cellular functions.

A starting point to explore a cell’s RNA–protein interaction network architecture is given by a systematic analysis of interactors at particular nodes or points of control. This can be approached from two angles—either the protein- or RNA-centered—that in principle provide a complementary view. Technically, the “protein-centric” approach relies on the ability to purify a particular RBP or accommodated complex thereof, followed by identification of the bound RNAs with DNA microarrays or high-throughput sequencing. Therefore, RBPs are usually isolated from cellular extracts with specific antibodies or via affinity purification of epitope tagged proteins. Such RBP immunoprecipitation followed by microarray or sequencing analysis of interacting RNAs (RIP-Chip/RIP-seq) was used early on for the recovery of native RNA–protein complexes under physiological conditions [11,12,13]; while more elaborate crosslinking immunoprecipitation (CLIP) [14] or photoactivatable ribonucleoside-enhanced crosslinking and immunoprecipitation (PAR-CLIP) [15], which involves the crosslinking of RNA–protein interactions in vivo upon exposure of cells to ultraviolet (UV)-light, enabled isolation of the RBP with bound RNAs under stringent conditions and also captures transient interactions. Furthermore, the implementation of partial RNA digestion allowed obtaining a footprint of the RBP binding site on the RNA (reviewed in [16]). These protein-centric approaches have found widespread application and determined the RNA targets as well as the binding sites of hundreds of RBPs in different organisms (e.g., [17,18]). Moreover, the integration of the data suggests the presence of highly coordinated and interconnected posttranscriptional regulatory networks. For instance, numerous RBPs interacting with mRNAs tend to bind to functionally related mRNAs, forming so-called post-transcriptional operons or RNA regulons for expression coordination [19,20].

In contrast to the rapidly progressing “protein-centric” approaches, the establishment of global “RNA-centric” approaches has lagged behind for some time. Thereby, a distinction can be made between (i) the identification of proteins binding to an entire population of RNAs, such as polyadenylated (poly(A)) RNA, or total RNA; and (ii) defining all proteins/RNA that interact with a specific RNA expressed in a certain cell-type or tissue. The first is aimed at gaining an unbiased identification of RBPs in a cell or organism. Early attempts used protein microarrays or recovered poly(A) RNAs from budding yeast *Saccharomyces cerevisiae* using oligonucleotide deoxythymidine (dT) beads to identify interacting proteins with MS [21,22]. While at limited sensitivity at that time, it uncovered novel and unconventional RNA-binders, such as metabolic enzymes and proteins of the vesicular transport system [21,22]. The introduction of in vivo crosslinking of RNA–protein interactions by UV-irradiation of cells further enabled the capture of poly(A) RNA and bound proteins with oligo-(dT) beads under highly stringent conditions, and combined with high sensitivity MS analysis has greatly enhanced the detection of RBPs (e.g., [23,24,25,26]). Commonly referred to as RNA interactome capture (RIC), these experimental studies revealed many new and unconventional RBPs including metabolic enzymes, heat shock proteins, DNA-binding proteins, kinases across diverse organisms, establishing a new area for characterization of novel biological functions (reviewed in [5,9,10]). Besides RIC and the related enhanced RIC (eRIC) [27], a variety of complementary approaches have been introduced since then to select RBPs crosslinked to RNA irrespective of its poly(A) status. This includes organic phase-separation approaches such as Orthogonal Organic Phase Separation (OOPS) [28], phenol-toluol extraction (PTEx) [29], and protein-Xlinked RNA eXtraction (XRNAX) [30], as well as click-chemistry based methods [31,32]. Furthermore, concordant mapping of protein binding sites through protease-assisted digestion consolidated and suggested new RNA-binding motifs, including intrinsically disordered regions [33,34,35]. For further reading about the diverse procedures to define RNA-binding proteomes (RBPome), the reader is referred to recent reviews on the topic [5,10,36,37].

Conversely, the systematic investigations of factors that interact with a particular RNA, which remained notoriously challenging because most RNAs have considerably low expression levels in cells, are much less well explored. Many laboratories currently implement more simple in vitro approaches, where for instance, an in vitro transcribed (IVT) “bait” RNA is coupled to beads and incubated with cell extracts to pull-down interacting proteins [38]. This approach bears the advantage of speedy testing of different RNA fragments and/or mutagenesis to localize protein interaction sites. However, this is an artificial system and the coassembled complexes may not represent authentic RNPs found in living cells for a number of reasons. For example, the synthetic RNA may not fold properly in vitro and lacks modifications, and nonspecific RNA–protein interactions could form during cell lysis and purification. Thus, in vivo approaches for biochemical characterization of endogenously formed RNP complexes remains a key goal towards gathering an understanding of the factors that combinatorially control the RNA’s fate in cells. Although challenging, substantial progress has been made towards this end during the last few years, mainly through techniques that implement antisense oligonucleotides (ASOs). In this review, I outline these recent developments, providing a brief overview of the methodological concepts for capturing crosslinked RNPs from cells via affinity purification. I also outline recent advances for the in vivo labeling of proteins that are in close proximity to a target RNA region using modified CRISPR/Cas9 systems.

## 2. RNA-Centric Approaches to Capture Selected RNAs—Overview and Concepts

**Figure 1:**
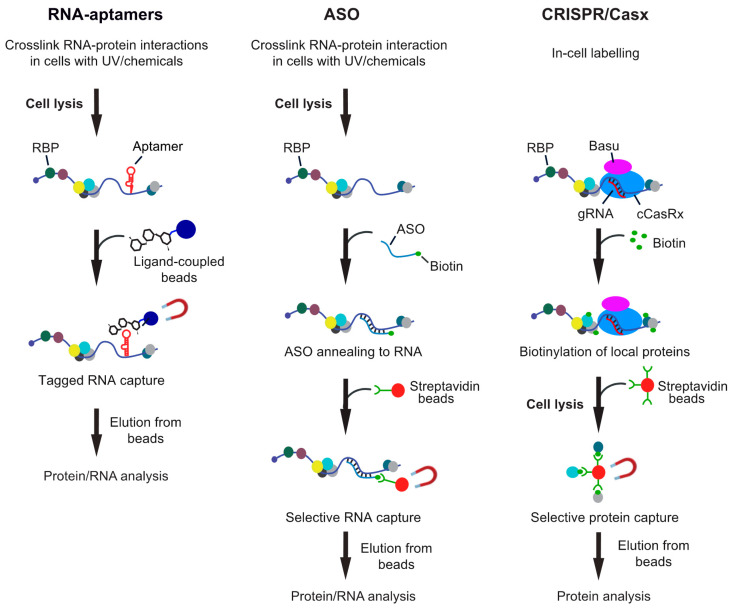
Overview of different RNA-centric approaches to isolate in vivo formed ribonucleoprotein (RNP) complexes. Left: Aptamer tagged RNAs are expressed in cells and captured from cell lysates with an aptamer binding ligand, which is coupled to beads. Shown are beads coupled with tobramycin, but protein ligands can also be employed. Middle: Antisense oligonucleotide (ASO)-based approaches rely on the hybridization of one or multiple biotinylated oligos with the target RNA at high selectivity. The annealed oligos are retrieved with streptavidin beads and eluted either with competitors (i.e., biotin or displacement oligos), heat or upon RNA digestion. Right: CRISPR-based approaches use gRNA-mediated targeting of an inactive CRISPR/Cas9 variant fused to a biotinylating enzyme (e.g., BASU) to the RNA of interest. Addition of biotin to the medium induces the biotinylation of proteins in close proximity, which can be recovered with streptavidin beads from extracts.

In principle, one can distinguish between in vitro approaches, where the “bait” RNA is usually coupled to beads and incubated with extracts for pull-down of associated proteins; and in vivo approaches, where RNA–protein interactions are stabilized in cells and then RNAs captured from cell lysates. Essentially, most biochemical procedures for isolation of specific RNAs can be applied in vitro and in vivo with modifications and either involve tagging the RNA of interest with an affinity aptamer and recovery with a high-affinity ligand coupled to beads, or the capture of native RNAs with modified ASOs (Figure 1). While the in vitro approaches have become common practice in many laboratories, in vivo analysis of interactors with particular RNAs remained challenging, mainly because many RNAs have low expression levels in cells and constitute only a tiny fraction of all RNA molecules. This is especially true in the case of lncRNA as many of them are expressed at extremely low levels in cells, such as the X-inactive specific transcript (*XIST*) lncRNA, where one copy in the nucleus accomplishes X-chromosome inactivation in females [39]. Thus, any biochemical approach for their isolation obviously needs a rather large amount of starting material for eventual detection of proteins or RNA interactors with MS or RNA-seq, respectively. Furthermore, the outcome of this analysis is highly dependent on the stringency of the protocol to reduce background. Therefore, irradiation of cells with UV-light or addition of chemicals such as formaldehyde to stabilize RNA–protein interactions in vivo are commonly used and enable purification of RNA with interacting proteins under stringent and/or denaturing conditions. On this line, one should be aware of the pitfalls of different crosslinkers that could bias the outcome of the analysis [29,36,37]. In brief, UV crosslinks induces emission of free radicals leading to irreversible covalent links by between nucleic acids bases (preferentially uridines) and close-by amino-acids of the interacting protein. The efficiency of crosslinking is relatively low (≈5%) and even less so for double-stranded (ds) RNA. Although often neglected, UV light can also induce protein–protein and DNA–protein crosslinks albeit likely at lower efficiency as compared to chemical crosslinkers (e.g., [40,41]). Finally, UV irradiation of cells could trigger cellular stress-responses that may cause rearrangement on post-transcriptional regulatory events. On the other hand, chemical crosslinkers, such as formaldehyde can be directly added to cells in culture and are therefore also applicable to tissues/organism that cannot be well penetrated with UV-light. While more efficient than UV, they have a higher propensity to introduce protein–protein and protein–DNA crosslinks. However, chemically induced crosslinks are reversible, which can make downstream applications more straightforward. In any case, the crosslinking needs to be fairly adjusted and optimized to minimize potential artefacts. On this line, recently developed CRISPR/Cas9 targeting approaches for exploration of local RNA–protein interactions represent an interesting in-cell labeling approach without the need of any crosslinker (see Figure 1).

## 3. Affinity Capture of RNAs via Aptamers

One commonly used concept for purification of RNP complexes involves the addition of an RNA aptamer tag to the RNA of interest, which permits capturing the tagged RNA with a high-affinity ligand (Figure 1). A variety of RNA aptamers are available, and the reader is referred to a recent review further describing RNA-aptamers and RNA labeling techniques [42]. Essentially, two classes of RNA aptamers are commonly used, either binding to small molecules or to proteins. Regarding the first, several RNA aptamers are available that bind with high affinity to aminoglycoside antibiotics, such as tobramycin and streptomycin [43,44]. These aptamers found early application to study the assembly of splicing complexes [45], identify proteins interacting with untranslated regions of mRNAs [46], or for the profiling of intron binding proteins and viral RBPs [47]. While mostly used in vitro, aminoglycosidic aptamers have also been applied in combination with RIC to recover in vivo formed RNP complexes [48].

A second class of aptamers includes short RNA hairpins that interact specifically with proteins, such as the coat proteins from the R17/MS2 bacteriophage [49], bacterial streptavidin S1 [50], the PP7 coat protein [51], the lambda bacteriophage anti-terminator protein N (or lambdaN peptide) [52], an engineered version of the Csy4 endonuclease [53], or artificial pentatricopeptide repeat (PPR) proteins [54]. While this class of RNA aptamers has been broadly used to globally determine proteins/RNAs interacting with the tagged RNAs in vitro (e.g., [52,55]; reviewed in [36,42,56]); the RNA aptamers and interacting proteins have also been coexpressed in vivo to recover endogenously formed RNP complexes from cell lysates through affinity capture [49,57,58,59]. For example, RBP purification and identification (RaPID) implemented the affinity purification of MS2 aptamer-tagged RNAs and detection of bound proteins and transcripts with MS and reverse transcription (RT)-PCR, respectively [49]. Specifically, the bacteriophage MS2 coat protein, which selectively interacts with the MS2 aptamer loop sequence (MS2L) was expressed as a fusion protein with GFP, allowing for in vivo localization studies, as well as the streptavidin-binding protein (SBP) for subsequent purification of formaldehyde crosslinked RNP complexes with streptavidin-conjugated beads [49]. In a related approach, termed MS2 in vivo biotin tagged RNA affinity purification (MS2-BioTRAP), histidine and biotin (HB)-tagged MS2 protein as well as MS2L tagged target or control RNA were coexpressed in cells, taking advantage of the tight association between tagged HB-MS2 and the RNA stem-loop tags for affinity purification of authentic RNA–protein complexes from UV irradiated cells under native or denaturing conditions [58]. To provide proof-of-principle, a 1.2 kb internal ribosome entry site (IRES) from lymphoid enhancer factor-1 (*LEF1*) mRNA was used as RNA target to unravel proteins associated with IRES function. SILAC-based proteomics identified about half the number of proteins under denaturing conditions (326 proteins) as compared to native conditions (535 proteins), suggesting that denaturing conditions removed indirect interactors and nonspecific binding factors. Using a non-IRES containing mRNA as control, 36 proteins were eventually identified as IRES binding proteins, which included known IRES binding factors but also novel interactors [58].

Noteworthy, an alternative approach to directly label the proteins interacting with a tagged RNA motif has been introduced recently [60]. RaPID (RNA protein interaction detection) integrates proximity-dependent protein labeling, based on the enzyme biotin ligase to identify the proteins that bind RNA sequences of interest in living cells [60]. Analogous to the above examples, the method involves the use of two components, an aptamer-tagged RNA and a fusion protein that interacts specifically with the tag. In particular, bacteriophage BoxB stem loops are integrated close to the RNA element under investigation; and a mutated biotin ligase from *Bacillus subtilis*, termed BASU, is expressed as a fusion with the lamdaN peptide that specifically interacts with BoxB stem loops. BASU is therefore tethered to the RNA-binding motif, thereby biotinylating proximal proteins within 10 nm upon growing of cells in media supplemented with biotin. The biotinylated proteins are then recovered with streptavidin-coupled beads under stringent conditions [60]. In this first instance, the method was used to evaluate proteins interacting with mutant RNA motifs and to define essential host proteins interacting with the UTRs of Zika virus RNA [60].

Overall, RNA-tags establish a relatively robust and versatile system for the recovery and characterization of RNP complexes in vitro with transferability to in vivo application and RNA localization studies. If incorporated in reporter system, aptamer tagged RNAs can be easily mutated and concomitantly enables monitoring of changes in gene expression and/or RNA localization. However, besides the need for cloning and transfection of cells with reporter plasmids, one drawback of these approaches concerns the ectopic expression of the protein or tagged RNA which may interfere with native RNP formation and/or mRNA maturation.

## 4. Capturing Endogenous RNAs with ASOs

The only biochemical approach that enables direct capture of endogenous RNA from any type of cells or tissues involves the annealing of ASOs with the RNA of interest (Figure 1). This approach was originally introduced to recover highly expressed RNAs and associated complexes, such as splicing small-nuclear RNPs [61] and telomerase RNAs [62,63]. Briefly, cell-free extracts were combined with biotinylated antisense modified 2′O-methyl RNA oligonucleotides, RNP complexes captured with streptavidin-coupled beads and finally released with an excess of displacement oligonucleotides. Despite their potential, ASO-based approaches remained fairly unattended for a while due to concerns regarding their limited sensitivities toward lower expressed RNAs, such as lncRNAs and/or mRNAs. However, several approaches have been introduced recently that along with the increased sensitivity of MS for protein detection enabled the capture and analysis of endogenously formed RNP complexes with high efficiency. In the following, I outline such recent examples that used ASOs for identification of interaction partners for (i) lncRNAs, (ii) viral RNAs, and (iii) mRNAs (Table 1).

### 4.1. Capture of lncRNAs with ASOs

ASO-based approaches to capture lncRNAs were originally developed to identify the genomic binding sites (reviewed in [78]). This included capture hybridization analysis of RNA targets (CHART) [79] and chromatin isolation by RNA purification (ChIRP) [80], both using chemical crosslinking with formaldehyde or glutaraldehyde, respectively; while, RNA affinity purification (RAP) applied UV irradiation of cells to induce crosslinks with RNA [81]. Commonly, these procedures used several biotinylated capture DNA oligonucleotides, which hybridize to the RNA of interest to isolate RNA-associated DNA or proteins from crosslinked cell-extracts with streptavidin-coupled beads (reviewed in [78]).

Recognizing their potential, the methods were further advanced and combined with MS analysis to identify interacting proteins. At first, in a proof-of-concept study, CHART-MS uncovered proteins bound to human *NEAT1* and *MALAT1* lncRNAs [64]. Therefore, several 25-mer DNA oligos were preselected by RNAse H test digestions to evaluate their accessibility to crosslinked RNAs, and the best oligo was used to capture these lncRNAs from 100 million formaldehyde-crosslinked MCF-7 and BJ cells. Besides hundreds of genomic trans-sites for both lncRNAs that strongly overlapped with active gene regions, 50–60 proteins were found enriched with each lncRNA [64]. A large fraction of those proteins was known to localize to nuclear speckles and paraspeckles and matched to a high extent the genomic colocalization of the lncRNAs.

At the same time, three independent studies used a “tiling” approach to investigate the *XIST* lncRNA interactome in different cell-types [65,67,68]. Applying RAP-MS, the *XIST* lncRNA was induced and isolated with bound proteins from nuclear extracts derived from 200–800 million UV crosslinked SILAC-labeled mouse embryonic stem cells (mESCs) using an array of long (90-mer) biotinylated DNA oligonucleotides covering the entire transcript [67]. After elution of proteins from beads with Benzoase (a nuclease from *Serratia marcescens*), 10 proteins were identified that reproducibly associated with *XIST* RNA over background levels determined from *U1* small nuclear RNA (snRNA) control affinity isolations with quantitative MS. Follow-up studies using siRNA-mediated knockdown of the interacting proteins showed that three proteins were required for transcriptional silencing (i.e., SHARP, LBR, and SAF0a) and that interaction of *XIST* with SHARP promotes the recruitment of other factors like SMRT and HDAC3 that deacetylate histones and thereby exclude access of polymerease II to the X-chromosome [67].

Substantially more proteins were identified with ChIRP-MS, possibly due to the implementation of chemical crosslinking of cells with 3% formaldehyde for a relatively long period (30 min) [65]. Initially, the method was tested for capturing highly expressed small-ncRNAs with oligonucleotides specifically targeting, *U1* and *U2* snRNAs, identifying 418 and 370 interacting proteins from HeLa cells, respectively; including proteins involved in splicing and pre-mRNA biogenesis, indicating the suitability of the approach. While only one oligo was used to capture these snRNAs, *XIST* was recovered from engineered mESCs with 43 20-mer DNA probes bearing a biotin triethyleneglycol (TEG) moiety at the 3′end, annealing along the entire mouse *XIST*. RNPs were liberated from beads by gentle biotin-elution to minimize contamination from nonspecific binders and samples subjected to label-free liquid chromatography (LC)-MS/MS analysis [65]. In total, 81 proteins were identified above background (2-fold enriched compared to small-ncRNAs (*U1*, *U2*, *U3*) affinity isolations) with known roles in chromatin modification, nuclear matrix, and RNA remodeling pathways; among them 14 proteins previously known to interact with *NEAT1* and *MALAT1* lncRNAs. Further functional characterization of interactors suggested that hnRNPK, a well-known RBP, participates in *XIST*-mediated gene silencing but not in localization, while SPEN is required for gene silencing. Collectively, the study concluded that *XIST* engages with proteins in a modular and developmentally controlled fashion to coordinate chromatin spreading and silencing. Noteworthy, the ChIRP-MS approach was used later to identify interactors of *MALAT1* [66]. Considering U1 affinity capture isolates as a control, 23 of the identified 970 proteins were considered to be specifically bound to *MALAT1* as compared to *U1* and probe-free control isolates. Additionally, follow-up studies validated the results and suggested that *MALAT1* binds to and, as a consequence, inactivates the prometastatic transcription factor TEAD, preventing the associating with its coactivator YAP and target gene promoters [66].

A third approach, termed ‘identification of direct RNA interacting proteins’ (iDRiP) was used to isolate the proteins interacting with *XIST* from UV-irradiated female mouse fibroblast expressing physiological levels of *XIST* RNA [68]. Here, nuclei were prepared and chromatin was solubilized by DNase I digestion, then *XIST*-specific complexes recovered with nine complementary 25-mer DNA oligonucleotides spaced across the entire 17 kb long RNA. Essentially, MS analysis revealed 80 proteins that were at least 3-fold enriched with *XIST* RNA over control samples obtained by recovery of abundant cytoplasmic and nuclear RNAs (*U6*, *Jpx*, and *18S* rRNA). While some previously known interactors for *XIST* were identified, the analysis revealed new proteins, many of them part of epigenetic complexes, such as cohesion complex proteins, histone modifiers, chromatin remodeling factors, topoisomerases, nucleoskeletal and matrix proteins.

More recently, Spiniello and colleagues introduced hybridization purification of RNA–protein complexes followed by mass spectrometry (HyPR-MS), which enables the analysis of interactomes of multiple RNAs in a single experiment [69]. In this regard, HyPR-MS allowed for the simultaneous and selective isolation of interactomes of the lncRNAs *MALAT1*, *NEAT1*, and *NORAD* from prostate cancer PC3 cell lysates. As compared to the “tiling” approaches, only 2–3 biotinylated capture oligonucleotides complementary to each lncRNA were mixed for capturing target RNA–protein complexes from formaldehyde crosslinked PC3 cell lysates with reasonable efficiency (up to 35%). Importantly, HyPR implements specific displacement oligonucleotides to release captured complexes from beads in a so-called ‘toehold-mediated release strategy’, which could increase selectivity towards particular RNA–protein complexes. As a result, 127, 94, and 415 interacting proteins were identified with label-free LC-MS/MS from *MALAT1*, *NEAT1*, and *NORAD* affinity isolates. Besides previously known interactions that served as an internal validation step, many novel interactors with functions related to documented features of the associated lncRNAs were discovered, including histone modifiers and transcriptional regulators. All interactomes were enriched for RBPs: while nuclear *MALAT1* and *NEAT1* were enriched for nuclear proteins, *NORAD*, a cytoplasmic lncRNA, was enriched for cytoplasmic proteins, confirming selectivity of underlying interactors in regard of the cellular compartmentalization. Interestingly, 20 proteins interacting with *MALAT1* were previously identified with CHART-MS [64], corroborating the previously observed limited overlap between different methodological approaches.

In conclusion, these studies showed the applicability of ASOs for capturing even low expressed lncRNAs. Nevertheless, the different approaches revealed only limited overlap of identified proteins, indicating that the screen is not exhausted yet and further technical improvement may be required. The “tiling” approach may be particularly beneficial for capture of very long RNAs such as the 17 kb long *XIST*, where shearing could break the RNA during preparation, thus making aptamer tags or using only a few oligos placed in particular locations less attractive. Nevertheless, the “tiling approach” bears limitation for analysis of distinct transcript isoforms or association of particular regions of interest within a given RNA, which could benefit from using fewer oligos specifically designed to capture certain transcript isoforms. Finally, the use of multiple oligos substantially increases the probability for cross-hybridization with unrelated RNAs. Nevertheless, these studies revealed new facets for understanding the function and molecular mechanisms of *XIST* and other lncRNAs, and the reader is referred to a recent review further highlighting these developments [82].

### 4.2. Capture of Viral RNAs

Similarly, ASO-based approaches were used to identify interaction partners of viral RNAs, which could provide information about key RBPs that could contribute to viral replication and proliferation. An early study used 39 nts long biotinylated DNA molecules that annealed with synthetic 3′ nontranslated region (3′NTR) of the hepatitis C virus (HCV) (+) genome as bait to capture interacting cellular proteins from hepatocyte extracts [83]. A total of 79 cellular proteins were identified with MS, most of which were RBPs with some having roles in HCV replication. However, it was realized that some proteins also interacted with the oligo-DNA probe alone, which had to be removed from the list as nonspecific binders. Bearing these limitations in mind, the same group formulated an interesting strategy that captured the replicating HCV (+) strand genome in situ for identification of associated cellular and viral factors. Related to peptide-nucleic-acid-assisted identification of RBPs (PAIR), a protocol that implements peptide nucleic acid (PNA) probes with cell-penetrating peptides to gain entry into cells and hybridize to the viral RNA [84], a sequence-specific and biotinylated PNA-neamine conjugate was used to target *HCV* RNA through hybridization with the subgenomic HCV (−) RNA in MH14 host cells (derived from Huh7 hepatoma cells) [72]. This interesting approach led to the recovery of three viral proteins (NS5B, NS5A, and NS3–4a protease-helicase) and 83 cellular proteins, which were enriched for translation factors and other RBPs as well as transcriptional regulatory and metabolic enzymes. Further siRNA-mediated gene silencing of selected proteins revealed enhanced or repressed HCV replication/translation. The study was unique as it used PNA, which is an unnatural DNA mimic with no sugar phosphate backbone, which is likely not recognized by cellular proteins and, therefore, being highly stable and resistant to cellular nucleases and proteases and reducing background signal due to nonspecific binding. Furthermore, the PNA-neamine conjugate efficiently penetrates cells and binds to its target RNA in the cytosol quasi irreversibly, which was taken in advantage to recover in vivo captured RNPs from the cell lysate [72]. Nevertheless, the target RNA itself may still be cleaved and transfection of PNA-based oligos may induce secondary effects that need to be monitored. Furthermore, since no crosslinking procedure was applied, some rearrangement of RNPs may have occurred during cell lysis and purification.

Thiouracil cross-linking mass spectrometry (TUX-MS) applied a modification of the RIC procedure to capture poly(A) RNAs using immobilized oligo(dT) to identify proteins bound to the polyadenylated poliovirus (PV) RNA genome in infected cells [73]. Thereby, HeLa cells stably expressing uracil phosphoribosyltransferase (UPRT) can incorporate 4-thiouridine (4sU) into newly synthesized RNAs if cells are grown in media containing 4-thiouracil (4TU). The addition of actinomycin D (ActD), a compound that inhibits cellular transcription but not viral polymerases, resulted in the labeling of the viral RNA, which was then crosslinked to proteins in vivo with UV at 365 nm and followed by the capture of poly(A) RNA with oligo(dT) coupled beads. This approach identified 15 previously known plus an additional 66 putative host proteins that bind to the PV RNA during infection in HeLa cells, a majority of them affecting viral amplification. These results showed that TUX-MS is a valuable tool to identify host factors for polyadenylated viral RNAs [73]. However, the method requires engineered cell-lines to express the labeling enzymes and thus limits the scope of host cells for investigation.

A “tiling” ASO approach was further used to purify dengue virus (DENV) RNPs from infected hepatocytes Huh7 cells [74,85]. Since the *DENV* RNA is not polyadenylated, the common strategy for capture of cellular polyadenylated RNAs was modified. Essentially, 10 DNA oligos (between 17 and 30 nts in lengths) containing a TEG spacer between the biotin moiety and the DNA at the 3′ end were used to recover the viral RNA from UV-crosslinked samples using streptavidin beads under stringent conditions. Similar to CHART-MS, selection of best ASOs for recovery of viral RNAs was adjusted through the testing of several antisense DNA oligos in RNase H mapping assays for their suitability to anneal with viral RNA [85]. Using mock-infected cells as a control, MS analysis revealed 12 host proteins bound to *DENV* RNA in vivo applying stringent selection criteria (20-fold above control isolates). Finally, siRNA mediated gene silencing followed by analysis of viral replication showed that at least half of the tested proteins likely play a role in virus replication [74]. Of note, recently a tiling approach using 120-mer biotinylated DNA baits specific to conserved regions of the *DENV* RNA genome was used to capture the viral RNA from total RNA [86]. It would be interesting to see whether it could be combined with MS to identify interacting proteins as well.

Finally, the above-mentioned HyPR-MS approach was also applied to enrich spliced full-length HIV RNA–protein complexes preserved in vivo by formaldehyde crosslinking to identify interactors from infected human cells [70]. Only one 30 nts long oligo complementary to unspliced *HIV-1* RNA was apparently sufficient to fairly-well recover the viral replication-deficient RNA from cell extracts (35% efficiency). MS analysis identified 189 proteins that were enriched with *HIV-1* RNA as compared to scrambled control oligo isolates (FC > 2.2, *p*-value < 0.05), among them 90 proteins that were previously known to impact HIV replication. Furthermore, siRNA-mediated knockdown of several proteins revealed changes in HIV expression, suggesting biological implications in HIV life-cycle [70]. Thus, the considerable overlap of previously identified HIV-related host factors as well as observed changes in HIV expression upon knockdown of several candidates supported the ability of HyPR-MS to correctly identify RNA-interacting proteins.

In conclusion, several ASO-based approaches have been successfully applied to recover viral RNAs from infected cells. While some studies used multiple ASOs for recovery of the RNA, this may not be necessarily required; and UV-crosslinking generally disclosed less interaction partners compared to chemical crosslinking procedures as could be expected.

### 4.3. Specific mRNA Capture

To date, only a few studies have attempted to recover selected native mRNAs with bound proteins/RNAs. One of the first attempts used biotinylated DNA oligonucleotides to isolate mRNAs from formaldehyde crosslinked cell extracts to detect microRNAs that could modulate the mRNA’s expression [87]. While the study identified and validated several miRNAs modulating the expression of the three different mRNAs under study (alpha-1 trypsin, interleukin-8, and secretory leucoprotease inhibitor) the associated proteins have though not been analyzed.

To analyze the proteins interacting with particular mRNAs in vivo, we have recently introduced a tandem RNA isolation procedure (TRIP) to capture selected cytoplasmic mRNAs [75,88]. Essentially, TRIP is a two-step procedure that relies on the purification of polyadenylated mRNAs with oligo(dT) beads from UV-crosslinked cells, followed by the capture of specific mRNAs with 3′-biotinylated 2′-O-methylated RNA ASOs that are recovered with streptavidin-coupled beads. TRIP was tested for isolation of in vivo crosslinked mRNP from yeast, nematodes, and human HEK293 cells. While RNP complexes were recovered at relatively good efficiency (up to 70%), the two-step procedure adds selectivity by reducing contamination from highly expressed ncRNAs and biotin binding proteins. The initial proof-of-concept study confirmed interaction of proteins with the target RNA validated by immunoblot analysis [75,88]. Noteworthy, a recent variation of the procedure implementing a tobramycin (tob) aptamer for the second step was used to profile the changes inferred on mRNPs for tumor suppressor *p27/CDKN1B* 3′UTRs in cisplatin treated cells [48]. While 54 proteins were enriched with the reporter mRNAs as compared to control isolates (>2.5-fold enriched), 16 proteins changed their RNA association in drug-treated cells at least two-fold. SiRNA-mediated knockdown of several of those RBPs affected expression of *p27* mRNA upon cisplatin (CP) treatment, and knockdown of KHSRP enhanced the sensitivity of MCF7 adenocarcinoma cancer cells to CP treatment [48]. Noteworthy, this study was the first to report the rewiring of RNA–protein interactions on a specific mRNA upon changing conditions, revealing RBPs as potential targets for modulation of cancer-drug sensitivity.

Like TRIP, ‘specific RNP capture’ also implements nuclease resistant modified ASOs that have increased affinity towards RNA targets and reduces background binding [76]. In particular, the target RNA was recovered from extracts by hybridization with antisense locked nucleic acids (LNA). Thereby, the 20-mer LNA oligonucleotide contained a 3′extension with a flexible C6 linker and a primary amine enabling direct covalent coupling to magnetic beads. While the coupling may impinge on the accessibility of the probe for hybridization with the target RNA—possibly posing a problem for isolation of larger RNPs—the covalent coupling of oligos to beads withstands high salt concentrations and chaotropic detergents, supporting efficient reduction of background signals. The protocol was used to identify several RBPs interacting with reporter Renilla Luciferase (RLuc) mRNAs containing *Drosophila* Sex-lethal (Sxl) binding motifs in vitro, and for exploring the repertoire of proteins binding to 18S and 28S ribosomal RNA (rRNA) from UV-crosslinked Hela cells in vivo [76]. While rRNAs are highly expressed in cells (up to 10 million ribosomes per eukaryotic cell), the approach could potentially be suited for the recovery of lower expressed endogenous mRNAs or lncRNAs as well.

Two recent studies have successfully implemented short unmodified DNA oligonucleotides to capture mRNAs from cells. Applying HyPR-MS, *c-myc* mRNA, coding for one of the best studied oncogenes, was captured from formaldehyde crosslinked K562 cells [71]. Therefore, two DNA oligonucleotides were used to ensure uniform capture of the full *c-myc* transcript, while a scrambled oligo was used as control. In total, 229 proteins were found to be associated with *c-myc* compared to control samples (≥5-fold), including several RBPs previously known to interact with the mRNA. It also revealed novel interactors and comparison with published CLIP/RIP data strongly suggested those being bona fide interactors [71]. Conversely, vIPR (in vivo Interactions by Pull-down of RNA) used a tiling approach, using multiple 3′-biotinylated TEG linked 20-mer oligonucleotides to recover GFP-tagged *gld-1* mRNA from the nematode *Caenorhabditis elegans* [77]. The study also compared different crosslinking procedures, suggesting that UV-crosslinking compared to chemical crosslinking with 2% paraformaldehyde recovered some different RBPs at different efficiencies. For instance, RBPs binding to dsRNA were underrepresented in UV-crosslinked samples. The method appeared to be highly efficient and selective, recovering known interactor with 3′UTR sequences of *gld-1* in *C. elegans*. In addition, small RNA sequencing recovered known and biologically relevant miRNA interactors, revealing miR-84 as specific regulatory of *gld-1* transcripts. In conclusion, vIPR seems to be well-suited for in vivo applications; however, the implementation of many DNA oligonucleotides needs careful evaluation for each transcript because of potential cross-hybridization. Furthermore, the direct capture of RNP complexes with streptavidin beads from extracts may recover prominent biotin binding proteins depending on the type of cells/tissues.

## 5. CRISPR-Cas Based In Vivo Targeting Approaches

The recently developed CRISPR-based RNA targeting systems provide a new option to directly label RBPs in living cells without the need for crosslinking procedures [89,90]. For instance, RNA-United Interacting System (CRUIS) captures RNA–protein interactions in living cells by combining the power of CRISPR and PUP-IT to frame a proximity targeting system [89]. Specifically, the dead RNA-guided RNA targeting nuclease LwaCas13a (dLwaCas13a) is used as a tracker to target specific RNA sequences, while the proximity labeling enzyme PafA fused to dLwaCas13a labels any surrounding RBP. After in vivo labeling, the labeled biotinylated proteins are enriched from cell-free extracts with streptavidin-conjugated beads and identified with MS. Testing the system with *NORAD* identified 51 candidate proteins, six of those were previously reported to interact with *NORAD*, including proteins involved in splicing and mRNA processing. Furthermore, the approach was also used to capture the *p21* mRNA interactome, showing adaptability for this application to different types of RNA. Likewise, CRISPR-assisted RNA–protein interaction detection method (CARPID) leverages CRISPR-CasRx-based RNA targeting and proximity labeling to identify binding proteins of specific lncRNAs in the native cellular context [90]. Once more, the method was applied to identify proteins interacting with *XIST* as well as other lncRNAs such as *DANCR* and *MALAT1*. Essentially, the method uses two different guide RNAs (gRNAs) to tether cleavage incompatible CRISPR fused to the biotin ligase BASU to the RNA target, which then biotinylates proteins in close proximity. The study found 73 *XIST*-interacting proteins that were significantly enriched in at least one set of gRNAs over controls, with 23 proteins discovered with at least two different sets of gRNAs.

The CRISPR based systems allow the labeling of proteins that interact with (or are in close proximity to) a specific RNA fragment in living cells, alleviating some risk of RNA degradation through processing of samples i.e., extract preparation for biochemical purifications. Furthermore, and in particular with CRUIS, the RNAs were left at natural expression levels. Although promising, there are some drawbacks with these techniques. For instance, it is not yet fully clear how well RBPs or indirect interactors are labeled, and what the in-cell spacing range apart from the assembly of the gRNAs may be for detection of interacting proteins. Essentially, the proximity ligation approach provides a snapshot of the proteins within a certain distance to the CRISPR-attached ligase without discriminating between direct or indirect interactors with RNA. Furthermore, it is difficult to predict gRNA specificity and off-target effects may apply. Finally, the CRISPR complex is relatively large and upon assembly could affect the local structure of the target RNAs and compete/synergize with interactors. In conclusion, while the in-cell methods provide new and valuable tools to study local RNA–protein interactions in cell-culture models, further studies are needed to learn more about its reliability and the interpretation of the data.

## 6. Conclusions

As RNA-centered investigations are becoming increasingly popular to characterize the dynamics of the RBPome, methods to study the protein architecture on particular RNAs are now also becoming feasible. Besides a variety of different aptamer-based RNA-capture approaches, substantial progress has been made implementing ASOs, which is the sole approach for isolation of unmodified and in vivo formed RNPs. The “tiling” approaches seem to achieve very efficient recovery of the target RNA (between 70% and 90%) but specificity could be compromised through cross-hybridization and limitations apply for analysis of transcript isoforms. Several studies have also accomplished a fairly good recovery of target RNAs with only one to three ASOs, minimizing potential contamination through off-targets and may be the preferred route for investigation of “shorter” RNAs. While most studies used either way—UV or chemicals—for crosslinking RNA–protein complexes in vivo, only a few side-by-side comparisons have been done (e.g., [77]). Usually, UV-based studies revealed a lower number of interactors, adding to the common belief that UV irradiation of cells is more selective to uncover direct RNA–protein interactions than chemical crosslinking, which may reveal the “wider-sphere” of interactors; however additional side-by-side comparisons would be required to make conclusive statements.

Generally, the currently developed methods to investigate in vivo formed RNA–protein interactions may now find firm adaptation for studying the dynamic rearrangement of RNPs on particular RNAs under stress conditions, during development and in disease. Furthermore, studies of the RNA assembled factors across different cellular compartments may become feasible (e.g., RNA maturation). Finally, as more quantitative data will become accessible, it could pave the way to model regulatory networks for prediction of functional output. Currently, we are just at the starting point for a new journey along the RNA that will certainly lead to many exciting discoveries and fundamental insight into RNA biology, and ultimately lead to a better understanding of their cellular function in health and disease, thereby offering new options for therapeutic intervention.

## Figures and Tables

**Table 1:** ASO-based RNA-capture approaches.

Method	RNA Type	Target RNA	Crosslinker; ASO Probes	Ref.
Chart MS	lncRNA	*NEAT1, MALAT1*	FA; 25-mer DNA, 3′TEG-biotin	[64]
ChIRP-MS	lncRNA	*XIST*	FA; 43 × 20-mer DNA, 3′TEG-biotin.	[65]
	lncRNA	*MALAT1*	FA; 32 × 20-mer DNA, 3′TEG-biotin	[66]
RAP-MS	lncRNA	*XIST*	UV; 142 × 90-mer DNA, 5′-biotin	[67]
iDRiP	lncRNA	*XIST*	UV; 9 × 25-mer DNA, 3′TEG-biotin	[68]
HyPR-MS	lncRNA	*MALAT1, NEAT1, NORAD*	FA; 2–3 × 25–30-mer DNA, 8-nts toehold, 3′-biotin	[69]
	virus	*HIV*	FA; 30-mer DNA, 8-nts toehold, 3′-biotin	[70]
	mRNA	*c-myc*	FA; 2 × 25-mer DNA, 8-nts toehold, 3′-biotin	[71]
PAIR (like)	virus	*HCV(-)*	15-mer biotinylated PNA oligo	[72]
TUX-MS	virus	Polio	UV; oligo(dT)_25_ beads	[73]
n.s.	virus	*DENV*	UV; 10 × 17–33-mer DNA, 5′-TEG-biotin	[74]
TRIP	mRNA	*p27, gld-1, Pfk2*	UV; 21–25-mer 2′O-Met RNA, 3′-biotin	[75]
Specific RNA capture	mRNA rRNA	*Rluc-sxl (in vitro)* *18S*	UV; 20-mer LNA with C6 amine-linker	[76]
vIPR	mRNA	*gld-1::gfp, gfp::lin-41*	UV, FA; 10 × 20-mer DNA, 3′-TEG- biotin	[77]
	mRNA	*gld-1, lin-41, alg-1*	UV; 10 × 20-mer DNA, 3′-TEG- biotin	

Abbreviations: FA, formaldehyde; n.s., not specified; TEG, triethyleneglycol; others are described in the text.
